# 931. The Impact of Point-of-Use Water Filters in Reducing Infections in Hospital Burn Units

**DOI:** 10.1093/jbcr/irag033.471

**Published:** 2026-04-06

**Authors:** Daniel R Mueller, Joi A McMillon

**Affiliations:** i3 Membrane, Seattle, WA; J.A.D. Infection Control Experts, Pembroke Pines, FL

## Abstract

**Introduction:**

Background: Patients with severe burns are susceptible to hospital-acquired infections (HAIs) due to impaired skin barriers and long hospital stays. The hospital water systems are significant reservoirs for disease-causing organisms such as *P. aeruginosa*, Legionella species, Acinetobacter Species, and other multi-drug resistant pathogens, which can lead to wound colonization, systemic infection, and death. Point-of-Use (POU) water filters are emerging as a potent solution in infection prevention, but their impact in burn units requires more evaluation.

Objective: This narrative review aims to evaluate the current literature on the impact of point-of-use water filters in hospital burn units as a targeted method for infection prevention and control, focusing on microbial contamination and patient outcomes.

**Methods:**

A comprehensive search was conducted on medical databases including PubMed, Embase and other grey sources. Studies focusing on microbial load reduction and clinical outcomes following installation of point-of-use water filters were included.

**Results:**

Available evidence demonstrates that use of validated POU water filters significantly reduces the contamination of burn units and the infection rates by gram-negative bacteria. In some instances, the infection rates declined to zero following installation of POU water filters. The studies also show a decline in water-associated outbreaks following installation of POU water filters. The available data, however, remain limited by differences in the filter designs used and standard maintenance protocols. Data linking point-of-use water filters to direct patient.

**Conclusions:**

POU water filters are an effective adjunct to the water infrastructure in burn units, and they are an important part of infection prevention strategies in hospitals. Installation of these filters represents a cost-effective mode of curbing water-borne disease spread in burn units, leading to better patient outcomes and overall cost reduction in the treatment of severe burns patients.

**Applicability of Research to Practice:**

Translation of POU water filtration research into practice demonstrates feasibility and impact in preventing waterborne HAIs in burn patients. POU filtration represents an evidence-based, pragmatic intervention bridging research efficacy with real-world infection prevention needs.

**Funding for the study:**

N/A.

